# Exposed Pulps

**Published:** 1870-04

**Authors:** 


### Exposed Pulps.

C. E. Francis, D.D.S., N. Y. (*The Dental Cosmos*), believes that his success in the treatment of healthy pulp should encourage dentists to attempt the preservation of every healthy pulp. The course of treatment he has adopted in such cases is very simple, and has frequently been recommended by others as well as himself. It is this:—First, keep at bay the fluid of the mouth, and dry the cavity carefully with a bit of the softest prepared punk. Bathe it with pure creasote until pain subsides. Cut a small circular piece from a sheet of smooth note paper and place it directly over the wounded pulp, patting the edges down neatly and cautiously; then fill the entire cavity with a thin paste of oxychloride of zinc. If nicely done, the tooth is not likely to ache, the creasote coagulating the exudation from the pulp, and allaying irritation. If there is no *irritation*, there can be no *inflammation*, and the chances of success are more favorable. This should be guarded against, and for this reason he uses the paper cap, which not only prevents the chloride of zinc from touching the pulp, but is one of the best non-conducting agents of thermal changes that could be introduced in the same space. The tooth thus filled should remain in this condition a week or month, that the zinc may become quite hard; then remove the greater portion of it and refill with gold. A sufficient thickness should remain to protect the pulp from pressure during the process of consolidating the gold.—*Record.*

